# Promoting Mental Health in Unaccompanied Refugee Minors: Recommendations for Primary Support Programs

**DOI:** 10.3390/brainsci7110146

**Published:** 2017-11-01

**Authors:** Usama El-Awad, Atefeh Fathi, Franz Petermann, Tilman Reinelt

**Affiliations:** Center for Clinical Psychology and Rehabilitation, University of Bremen, Grazer Str. 6, 28359 Bremen, Germany; fathi@uni-bremen.de (A.F.); fpeterm@uni-bremen.de (F.P.); reinelt@uni-bremen.de (T.R.)

**Keywords:** refugees, mental health, adaptation, acculturation, intercultural competence, emotion regulation, motivation

## Abstract

During the last years, the number of refugees around the world increased to about 22.5 million. The mental health of refugees, especially of unaccompanied minors (70% between the ages of 16 and 18 years) who have been exposed to traumatic events (e.g., war), is generally impaired with symptoms of post-traumatic stress disorder, depression, and anxiety. Several studies revealed (1) a huge variation among the prevalence rates of these mental problems, and (2) that post-migration stressors (e.g., language barriers, cultural differences) might be at least as detrimental to mental health as the traumatic events in pre- and peri-flight. As psychotherapy is a limited resource that should be reserved for severe cases and as language trainings are often publicly offered for refugees, we recommend focusing on intercultural competence, emotion regulation, and goal setting and goal striving in primary support programs: Intercultural competence fosters adaptation by giving knowledge about cultural differences in values and norms. Emotion regulation regarding empathy, positive reappraisal, and cultural differences in emotion expression fosters both adaptation and mental health. Finally, supporting unaccompanied refugee minors in their goal setting and goal striving is necessary, as they carry many unrealistic wishes and unattainable goals, which can be threatening to their mental health. Building on these three psychological processes, we provide recommendations for primary support programs for unaccompanied refugee minors that are aged 16 to 18 years.

## 1. Introduction

Due to wars and disasters, the number of forcibly displaced people in 2016 increased to 65.6 million worldwide. These included 22.5 million refugees, of which 2.8 million were asylum seekers. Half of the refugees were underage and about 75,000 asylum applications were lodged by unaccompanied minors [1]. Germany received about half of these applications with 91.1% coming from male and 8.9% coming from female unaccompanied refugee minors (URM) [2]. More than 70% of all URM were between 16 and 18 years old [3]. Hence, supporting unaccompanied refugee minors (URM) is of importance to the German welfare and school system, especially for the youth welfare service as it assumes responsibility for URM by taking over the legal guardianship. The main function of the youth welfare service is to support children and adolescents in their development. However, in Germany, despite this responsibility and the large amount of refugees, there are no guidelines for programs that are research based, which target adaptation and mental health and cover different dimensions related to URM. Therefore, our aim is to introduce a framework that can guide practitioners in designing their programs. As several psychological research areas, e.g., clinical psychology, cultural psychology, and social psychology, contribute to the study of adaptation and mental health, the framework cannot rely on a coherent theory. Rather, it aims to represent the various research lines while drawing on cognitive, affective, and motivational concepts affecting mental health and adaptation, as these concepts are generally the focus of training (e.g., intercultural competence (IC), emotion regulation, goal setting and goal striving). 

For many years, concerns focused on URM’s well-being with regard to physical diseases and injuries that are caused by disasters and wars [4]. However, although many refugees endure physical injuries or hunger, far more suffer psychologically from traumatic experiences [5]. These experiences include, for instance, the experience of life-threatening events, physical maltreatment, sexual abuse, the loss of loved ones, separation from ones family against ones will, or the witnessing of violence towards others [6,7]. Especially, URM are at a higher risk of experiencing multiple traumatic events [7,8]. Hence, mental health problems, e.g., Post-Traumatic Stress Disorder (PTSD), Major Depression Disorder (MDD), Generalized Anxiety Disorder (GAD), Adjustive Disorders, Panic attacks and Somatization, have commonly been reported among URM [5,9,10,11]. However, the prevalence rates of diagnoses differ largely between studies, disorders, and types of measures. For instance, the prevalence rates of PTSD, as derived from self-reports on questionnaires, vary between 17% and 71%, while prevalence rates derived from clinical interviews vary between 20% and 30%. Similarly, prevalence rates for MDD vary between 9% in clinical interviews and 44% in self-reported questionnaires, while the prevalence rates for GAD vary between 4% in clinical interviews and 38% in self-report questionnaires. Hence, about 20% to 80% of URM show little psychopathological concerns and seem to be resilient [8]. In addition to being resilient from the beginning, the mental health of the majority of URM improves over time [12], thereby resembling the typical trajectories in adjustments to chronic stress or post traumatic events [13]. However, these positive adjustments might take some time as they have been reported in a 9-year follow-up study [12], while no improvements in the mental health of URM were observed within the first two years after their arrival in the host country [14,15].

## 2. Acculturation and Mental Heath

This high maintenance of mental health problems in URM during the first years after arrival might reflect post-migration stressors rather than the lasting impact of experienced traumatic events pre- or during the flight. Indeed, URM reported an increase in stressful life events within the first two years after arrival in the host country [14], which were mainly due to experiences of discrimination (e.g., feelings that others have prejudices about oneself or one’s country), insufficient housing conditions, or a general dissatisfaction with one’s leisure time [15,16]. In addition, there are cultural changes leading URM to the feeling of living in two different worlds [5]: People look different, wear a different wardrobe, speak a different language, eat different food, live by different religions, values, moral codes, or differ in their general way of thinking. According to Berry’s influential framework [17,18], this situation leads to processes of acculturation, i.e., changes in the perception of one’s own culture that take place due to contact with culturally dissimilar people or influences. Culture thereby refers to shared understandings or meanings kept by a specific group of people. The similarity between the host culture and the heritage culture determines the strength of the acculturation process during the adaption to the host culture [19]. If URM only need to learn some new aspects on how to deal within their host culture, the adaptation process will be rather smooth. However, if there are serious conflicts between appropriate behaviors in their heritage culture and in the host culture, or acculturation-specific daily hassles (e.g., perceived discrimination), this might result in acculturative stress [17,20], which usually manifests itself in mental disorders such as depression, anxiety, or in some cases in psychosis [21]. 

Indeed, URM reported an increased amount of both acculturation-specific and general hassles as compared to their peers from the host country and their peers with immigrant background but without flight experience [22,23]. Associations between daily hassles and symptoms of depression remained even after influences of traumatic events pre- or during the flight had been controlled [24,25]. In addition, longitudinal analyses revealed that the extent of acculturation-specific daily hassles predicted the trajectories in adaptation to traumatic events. A lower extent of daily hassles predicted resilient trajectories right from the beginning, while a higher extent of daily hassles predicted chronic trajectories as well as improved trajectories [26]. Furthermore, regarding the causal direction, these models supported an influence of daily hassles on depressive symptoms rather than an influence of depressive symptoms on daily hassles [26]. Hence, the elevated risk for daily hassles in URM cannot be explained by their traumatic experiences.

In addition to daily hassles, the amount of acculturative stress URM experience is influenced by their acculturation strategies [17,18]. Acculturation strategies are based on two components: (1) orientation (i.e., attitudes and activities) towards the host culture and (2) orientation towards the heritage culture. Based on these two orientations, four acculturation strategies can be derived: integration, assimilation, separation, and marginalization. Integration is defined by both an orientation towards the host culture and an orientation towards the heritage culture. In contrast, assimilation is denoted by an orientation towards the host culture, but no orientation towards the heritage culture, while separation is described by no orientation towards the host culture, but an orientation towards the heritage culture. Finally, marginalization is defined by both no orientation towards the host culture and no orientation towards the heritage culture [18]. Across several studies, integration has been regarded as the most favorable orientation in terms of acculturation and mental health. Adolescent refugees following an integrative orientation demonstrated a higher self-esteem, had more close friends, and were more accepted by their peers as compared to refugee adolescents following other acculturation strategies [27]. An integrative orientation was related to less depressed mood after controlling for traumatic experiences, however, no differences to an assimilation strategy were reported, whereas both separation and marginalization strategies were associated with an elevated depressive mood [25]. In addition, adolescent refugees applying a marginalization strategy were at a greater risk of experiencing daily hassles, indicating an interaction of acculturation strategies and post-migration stressors [25].

In addition, acculturation is a process that occurs over time. According to Berry [28], there are three distinguishable phases: contact with the host culture, conflict with the host culture, and adaptation to the host culture. Contact lies at the core of acculturation as the purpose, nature, duration, and permanence of contact affect the acculturation process. For instance, Berry [28] noted that if the contact with the host culture has no purpose and is rather random, then the contact will be short-termed. In particular, communication is remarkably important during the contact phase and needs to be constructive and without failures and misunderstandings as much as it is possible [28]. Therefore, sensitivity to cultural differences is a prerequisite for a successful intercultural communication and the basis for a more general intercultural competence [29]. 

## 3. Acculturation and Intercultural Competence

With significantly rising global diversity, intercultural competence (IC) is considered a significant aspect of acculturation, biculturalism, or multiculturalism [30]. It reflects the effectiveness of people’s communication and their activities together [29]. Therefore, IC can be described as the capability to appropriately and effectively carry out social interactions and communicate with people from various cultures based on one’s own intercultural knowledge, skills, and attitudes [31].

Kim [32], as the first one who examined the role of communication in the acculturation process, discussed that the communication environment is strongly linked to personal and social communication and can be considered as a significant determinant of acculturation. The frequency and intensity of interactions and communication with members of the host society has a remarkable influence on immigrants’ acculturation process. Hence, communication in the first phase of acculturation, when contacts to the host society are established, is crucial [33]. While involvement in their native ethnic community may foster the acculturation process of URM in the beginning, it may delay the acculturation process in the long-term. This is because the acculturation process mostly adopts and adapts to focal patterns and rules of communication in the host society. Moreover, the immigrants’ communication competence can facilitate all of the other aspects of adaptation and adjustment in the host culture, so that intercultural communication competence can be considered as both the fundamental process and the main outcome of the acculturation process [34]. During the acculturation process, IC develops from an unconscious incompetence to an unconscious competence [35], as sensitivity to cultural differences grows and, thereby, shapes the competence for intercultural communication [29].

Among other factors that affect the levels of IC, emotion regulation might be particularly important as especially empathy may enable URM to reach a level of unconscious competence behavior and adapt to intercultural differences [36,37] while emotion dysregulation has been shown to be detrimental for IC [38]. In addition, competencies in emotion regulation have been associated with better mental health in general.

## 4. Emotion Regulation and Mental Health

Emotion regulation can be described as a process of the person’s capability to evaluate, manage, experience, express and improve emotional reactions in a way that helps proper functioning [39]. It leads to a higher ability to appropriately respond to emotional experiences and can be seen as a dynamic process in which positive and negative emotions are being increased or decreased by using emotion regulation strategies [39,40]. Within Gross’ [39] Process Modell of Emotion Regulation, strategies are categorized by the time they first affect the process in which emotions are being unfolded. Antecedent-focused strategies differ from response-focused strategies and precede physiological or behavioral responding on emotions. These include the reappraisal strategy, which is leading to most positive outcomes by changing the way in which a situation is interpreted and decreasing its emotional impact [41]. It changes cognition by deciding on alternative meanings of previously selected situational aspects [39]. 

Emotion regulation strategies both facilitate the acculturation process and are generally helpful to deal with traumatic experiences [42]. For instance, there are experimental evidences that the execution of adaptive emotion regulation strategies such as reappraisal can significantly diminish rage and aggression [43,44]. In contrast, emotion dysregulation has been associated with impaired anger management [45,46,47,48]. 

Regarding refugees, they not only experience extreme fear or shame because of traumatic experiences, but also often have difficulties with emotion regulation and anger management [49,50]. In addition, in a study on formerly abducted adolescents, who had been living in Ugandese rebel camps, difficulties in emotion regulation were associated with exacerbated symptoms of PTSD or depression [51] and emotion dysregulation mediated the effects of traumatic experiences on symptoms of PTSD, depression, and anger [52]. An additional study on the relation of emotion dysregulation, post-migration living difficulties and PTSD revealed a partial mediation of the effects of post-migration living difficulties on symptoms of PTSD and depression through emotion dysregulation, as well as a complete mediation of the effects of post-migration living difficulties on anger [52]. Consequently, impaired emotion regulation might act as a mechanism empowering the connection between asylum seekers and refugees’ experiences and mental health outcomes [52]. The experience of extreme emotional distress in connection with trauma and post-migration living difficulties might force the refugees to use dysfunctional emotion regulation strategies and the lack of access to functional strategies may thus empower the association between post-migration living difficulties and PTSD and depression symptoms [52].

## 5. Goal Setting and Goal Striving and Mental Health

In addition to emotion dysregulation, post-migration living difficulties and the experience of traumatic events in refugees have been associated with difficulties in engaging in goal-directed behavior [52]. Upon their arrival in a host country URM have expectations towards the new country and goals that they like to achieve. They might expect living in peace, getting a good education, finding a well-paid job, or living in nice houses. However, finding a nice house is not always easy. It takes some time to get a place in a school or vocational school; educational degrees from home countries are not always accepted, and getting a well-paid job is rather difficult and sometimes impossible [53,54,55]. Thus, not all expectations or goals of URM can be met. Problems to disengage from unattainable goals, however, have been associated with depressive symptoms and a poorer mental health in general [56,57,58]. Indeed, unmet expectations regarding the host country have been reported both by clinicians treating Somali refugees in the United Kingdom and the USA [59] as well as Sudanese refugees in Canada [60] and have been related to psychological problems [60].

Several motivational strategies have been developed in order to help people setting attainable goals and striving for them. For instance, the strategy of mental contrasting (MC) connects people’s wishes with obstacles impeding their fulfillment [61]. During MC people name a wish and elaborate on it through imagining in detail the best possible outcomes associated with its attainment. Subsequently, people name an aspect of the reality, which hinders them in attaining their wish, and elaborate on it. This procedure leads people to align their wishes with their expectations of success. If expectations to accomplish one’s wish are high, people view the aspect of reality as an obstacle and form a strong association between the positive outcome and this obstacle, which leads to an enhanced goal commitment, energization, and goal striving. However, if expectations to accomplish one’s wish are low, people do not regard the impeding reality as an obstacle, no associations between the impeding reality and the positive outcomes are formed, and, consequently, goal commitment, energization, and goal striving are reduced [61,62,63]. Thus, MC leads to strengthened goal striving in the light of high expectations of success, but to weakened goal striving and goal disengagement in the light of low expectations of success. To further enhance goal striving for feasible goals, MC has often been combined with implementation intentions [64]. Implementation intentions are a specific form of plan in the form: If situations X occurs (when and where), I will perform behavior Y. 

## 6. Programs Supporting URM’s Mental Health and Adaptation

Despite the high number of resilient URM and factors influencing mental health other than pre-flight traumatic experiences, most clinical interventions addressing mental health of URM focus on PTSD [65]. However, therapeutical interventions in face-to-face settings are a limited resource and the need of cultural-sensitive translators for specific languages [66] further depletes the resource. Hence, psychotherapy should be reserved for severe cases, while support programs should also target other factors that are directly influencing mental health (e.g., emotion regulation strategies or strategies for goal setting and goal striving) as well as factors indirectly affecting mental health through the acculturation processes, e.g., intercultural competence [67].

In contrast, governmental or organizational programs mainly support acculturation processes. However, most programs focus on structural economic, educational, or health aspects, as for instance providing refugees with housing, schooling, or vocational education, or integration in the labor market [53,54,55,68]. One major prerequisite for integration in schools and especially in the labor market is language. Therefore, language courses are often offered by the public or non-profit organizations [69,70]. In addition, several states like Germany offer government-funded integration courses, which cover topics as the German legal system, history, important values of the society, as well as rights and obligations of residents and are sometimes directed at specific target groups as young adults or women [70]. 

However, these integration courses are generally only loosely based on psychological concepts that are known to influence acculturation (e.g., intercultural competence) and mental health. In addition, they often consider refugees as helpless individuals, who are not able to take care of their lives independently. Yet, on the contrary, refugees are mostly individuals with a strong intention to survive and pull through, who have come to the host country with their own wishes and goals [71]. In addition, while clinical programs tend to focus only on psychotherapy, integration courses ignore the mental health condition of refugees, and especially URM, at all.

Therefore, primary support programs, which are designed for groups of URM, should include both elements that directly aim to reduce sub-threshold psychopathologies as well as foster acculturation by integrating the URMs own goals. 

## 7. A Working Model for Developing Primary Support Programs for Adaptation and Mental Health of URM

**Figure 1:**
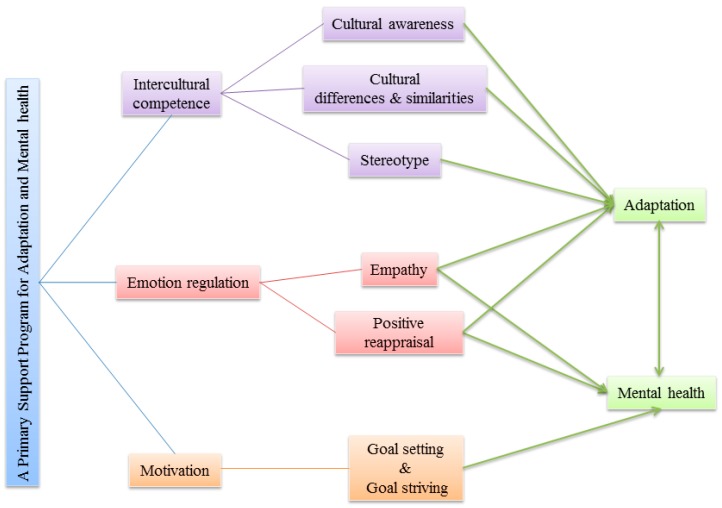
A working model for a primary support program for adaptation and mental health. A primary support program for unaccompanied refugee minors (URM) should include three main psychological elements: intercultural competence, emotion regulation and motivation. Training intercultural competence should include lessons on cultural awareness, cultural differences and similarities, and stereotypes in order to foster adaptation. Training emotion regulation should work on participant’s empathy and strategies of emotional acceptance and emotional modification. This should foster the adaptation of URM and their mental health. During the motivation training, URM learn to set realistic goals and to strive for them efficiently, leading to improvements in mental health.

The reviewed literature on mental health and acculturation in URM revealed several key constructs—intercultural competence, emotion regulation, goal setting, and goal striving—that root in different lines of psychological research. While intercultural competence is mainly discussed in cultural and organizational psychology, emotion regulation is a key construct in clinical psychology and developmental psychopathology, whereas goal setting and goal striving are primarily rooted in social and motivational psychology. Consequently, so far, no unifying framework exists that could guide the development of primary support programs for URM. Figure 1, therefore, depicts a working model how such a framework could look like. As intercultural competence rather draws on cognitive processes, while emotion regulation, as well as goal setting and goal striving reflect affective or motivational processes, respectively, the three processes might constitute rather independent ways to influence adaptation and mental health. However, in order to develop primary support programs for URM it would be critical that these three concepts—IC, emotion regulation, goal setting and goal striving—could actually be trained within the age of 16–18 years, the main age group of URM [3]. 

### 7.1. Training Intercultural Competence

As sensitivity to cultural differences is the basis for successful intercultural communication and IC in general [29], trainings of intercultural competence should include lessons on the concept of culture, cultural differences and similarities, stereotypes, adaptation skills, attitudes of respect and appreciation, and intercultural communication abilities. For instance, by giving knowledge about culture, URM will become more familiar with the norms, customs, cultural and social activities, the way of communicating and greeting, or the way of making friends in a host culture. Learning about cultural differences and similarities between refugees’ heritage culture and a host culture, enables URM to balance their own cultural values and the host society’s cultural values. In addition, learning about stereotypes that are related to different cultures, ethnicities and religions should help URM to understand other people’s reactions and behaviors by informing them how stereotypes can affect people’s reactions and expectations. Several training programs for IC have been developed for immigrants supporting a general effectiveness [72,73,74]. Hence, primary support programs for URM should include elements of IC. 

### 7.2. Training Emotion Regulation

Based on Gross’ model for emotion regulation [39], improving emotion regulation leads to a higher ability to appropriately respond to emotional experiences. It is especially empathy that is strongly linked to emotional competence [75], but training programs also focus on other strategies, as for instance on the acceptance of one’s own emotions or reappraisal. Several studies revealed positive effects of emotion regulation trainings as part of a trauma-focused cognitive behavioral therapy, improving clinical outcomes and reducing dropout rates [76,77]. Similar effects have been reported for patients with major depression [76], as well as for non-clinical children and young adolescents [78]. With regard to refugees, studies on Cambodian refugees performing culturally adapted forms of cognitive behavioral therapy [79] suggested that progression in emotion regulation ability successfully accelerates PTSD symptom reduction [80]. In particular, emotion regulation strategies of acceptance and mindfulness were beneficial [38]. 

Hence, as emotion regulation capabilities are generally reduced in refugees and better emotion regulation strategies foster mental health and the acculturation process, primary support programs for URM should include elements of emotion regulation.

### 7.3. Training Goal Setting and Goal Striving

Mental contrasting is a common strategy of goal setting and has been successfully applied to various wishes (interpersonal concerns, health, academic performance) and populations, including depressive patients [81] and children with externalizing disorders [82]. In addition, with regard to goal striving, II are one of the most successful strategies to initiate behaviour. They are common in health psychology [83], have even been utilized by children with externalizing problems [84,85], and meta-analytical effects are moderate to high [86,87]. Effects are especially high, when MC and II are combined [88]. However, most studies investigated the effects of MC, II, and MCII with regard to goal attainment and only few evidence has been reported for effects of II on goal disengagement [89].

Hence, as URM often carry unrealistic expectancies and wishes towards their life in the host country, primary support programs for URM should help them in setting realistic goals, disengage from unattainable or unrealistic wishes, and strive efficiently towards goal attainment. The strategies of MC and II might be suited for support programs for URM, in particular, as they have proven their value for different populations and goals, including children and adolescents, as well as people suffering from internalizing and externalizing problems. In addition, the strategies are easy to administer and learn. 

## 8. Discussion, Limitations and Recommendations for Practice and Research

Unaccompanied refugee minors are at a special risk for mental health problems due to traumatic experiences pre or during their flight as well as difficulties in the acculturation process. So far, support programs for URM either mainly focus on their traumatic experiences and resulting PTSD or on structural aspects as language and integration courses, integration into the schooling system or the labor market. However, these programs do not directly address psychological processes, which might offer URM resources, they might be able to use subsequently during their acculturation process. Primary support programs, which include elements of IC, emotion regulation, and goal setting and goal striving, therefore fill a gap. These elements are easy to integrate into the existing programs and can be applied in group settings, thereby reducing costs in terms of time or money. However, training material will need to be translated into the different languages of the URM currently coming into the host countries and validation studies should be carried out to ensure the material is culturally appropriate. Moreover, for larger groups it might be advisable to use translators and interpreters, who are more familiar with the refugees’ cultural backgrounds and have some experiences related to trainings or psychotherapies for refugees [66]. Due to the absence of a naturally-arising social support for URM, it is recommended to apply support programs, which connect them to the members of their own cultures and ethnicities. Such programs will help URM to receive social supports from both original and new cultures and subsequently reduce psychological difficulties [90].

While about 70% of the URM are between 16 and 18 years [3], it is unclear whether primary support programs for younger URM can rely on all three concepts. Both emotion regulation trainings and II have been successfully applied to children aged 9 to 12 years [78,84], however little is known on trainings of IC in this age group and none of concepts has generally been trained in even younger children. Hence, the appropriateness of training these concepts in younger children remains a question of future research.

In addition, longitudinal studies are needed in order to evaluate the effectiveness of these program elements and whether the elements affect adaptation and mental health independently of each other or are interrelated. Furthermore, future research needs to analyze the personal characteristics of URM or the program conductors, which might act as potential moderators for the success of primary support programs. Finally, the three described elements influencing mental health and adaptation are not exclusive and other constructs should be evaluated with regard to trainability and their supporting effects.
